# Dissociable functions of reward inference in the lateral prefrontal cortex and the striatum

**DOI:** 10.3389/fpsyg.2015.00995

**Published:** 2015-07-16

**Authors:** Shingo Tanaka, Xiaochuan Pan, Mineki Oguchi, Jessica E. Taylor, Masamichi Sakagami

**Affiliations:** ^1^Brain Science Institute, Tamagawa University, Machida, Japan; ^2^Institute for Cognitive Neurodynamics, East China University of Science and Technology, Shanghai, China; ^3^Graduate School of Brain Sciences, Tamagawa University, Machida, Japan

**Keywords:** lateral prefrontal cortex, striatum, reward inference, model-free learning, model-based learning

## Abstract

In a complex and uncertain world, how do we select appropriate behavior? One possibility is that we choose actions that are highly reinforced by their probabilistic consequences (model-free processing). However, we may instead plan actions prior to their actual execution by predicting their consequences (model-based processing). It has been suggested that the brain contains multiple yet distinct systems involved in reward prediction. Several studies have tried to allocate model-free and model-based systems to the striatum and the lateral prefrontal cortex (LPFC), respectively. Although there is much support for this hypothesis, recent research has revealed discrepancies. To understand the nature of the reward prediction systems in the LPFC and the striatum, a series of single-unit recording experiments were conducted. LPFC neurons were found to infer the reward associated with the stimuli even when the monkeys had not yet learned the stimulus-reward (SR) associations directly. Striatal neurons seemed to predict the reward for each stimulus only after directly experiencing the SR contingency. However, the one exception was “Exclusive Or” situations in which striatal neurons could predict the reward without direct experience. Previous single-unit studies in monkeys have reported that neurons in the LPFC encode category information, and represent reward information specific to a group of stimuli. Here, as an extension of these, we review recent evidence that a group of LPFC neurons can predict reward specific to a category of visual stimuli defined by relevant behavioral responses. We suggest that the functional difference in reward prediction between the LPFC and the striatum is that while LPFC neurons can utilize abstract code, striatal neurons can code individual associations between stimuli and reward but cannot utilize abstract code.

## Introduction

Reward prediction is paramount for learning behavior (Sutton and Barto, 1998; Schultz, 2006) and for decision-making processes in the brain (Rangel et al., 2008). Much research has shown that many brain areas are involved in reward prediction (Yamada et al., 2004; Knutson and Cooper, 2005; Padoa-Schioppa and Assad, 2006; Paton et al., 2006; Behrens et al., 2007, 2008; Hare et al., 2008; Hayden et al., 2008; Hikosaka et al., 2008; Haber and Knutson, 2010; Rushworth et al., 2011; Levy and Glimcher, 2012; Garrison et al., 2013). The basal ganglia and multiple sub-areas in the prefrontal cortex especially play important but different roles in the reward prediction process (Watanabe, 1996; Hollerman et al., 1998; O’Doherty et al., 2003; Roesch and Olson, 2003; Samejima et al., 2005; Hare et al., 2008; Hikosaka and Isoda, 2010; Diekhof et al., 2012). Several fMRI studies have demonstrated the importance of both the lateral prefrontal cortex (LPFC) and the striatum in the basal ganglia for reward prediction and have compared the functional difference in reward prediction between them (McClure et al., 2004; Tanaka et al., 2006; Kahnt et al., 2011). Some studies in monkeys have also directly examined neuronal activities in the LPFC and striatum, providing results that suggest that both areas are involved in the learning of stimulus-reward (SR) associations and that both represent positive and negative reward prediction (Pasupathy and Miller, 2005; Kobayashi et al., 2007; Histed et al., 2009; Asaad and Eskandard, 2011; Pan et al., 2014). Because of the neuroanatomical and pharmacological differences between the cerebral cortex and basal ganglia, the likely functional differences between the LPFC and the striatum can be predicted. For example, numerous discussions have been performed about the functional differences between the prefrontal cortex and the striatum for the learning of behavior in the frameworks of goal-directed/habit learning (Balleine and Dickinson, 1998; Killcross and Coutureau, 2003; O’Doherty et al., 2004; Tricomi et al., 2009; Balleine and O’Doherty, 2010; McNamee et al., 2015).

**FIGURE 1:**
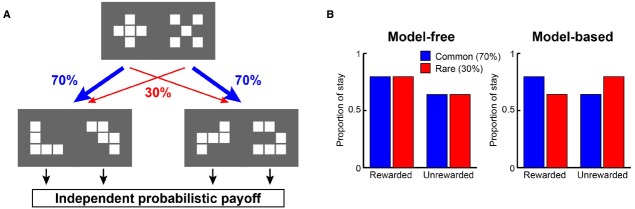
**A strategy to examine model-free and model-based learning processes. (A)** The two-stage Markov decision task described in Daw et al. (2011). The choice of the first stage led predominantly (70%) to one of the two pairs of options and rarely (30%) to the other pair. The probability of payoff associated with the four second-stage options was changed independently according to a Gaussian random walk protocol. **(B)** A model-free (simple reinforcement learning) strategy predicts higher stay probability in the first stage choice on the next trial after a rewarded present trial than after an unrewarded present trial, regardless of whether the present first stage choice led to a common or rare transition. On the other hand, a model-based strategy predicts higher stay probability in the first stage choice on the next choice after a rewarded present trial than after an unrewarded present trial in a common transition case, and after an unrewarded present trial in a rare transition case.

Recently, from the viewpoint of computational theory, vigorous discussion has emerged about the functional differences in the learning of behavior between the LPFC and the striatum. In particular, the hypothesis of Daw et al. (2005) which relates the difference between “model-based vs. model-free” processes to the difference in functions of the LPFC and striatum, is supported by the results of studies on humans and primates (Joel et al., 2002; Bunge et al., 2003; Doya, 2008; Maia, 2009; Rygula et al., 2010; Beierholm et al., 2011). According to this hypothesis, while the model-free process allows reward prediction to be achieved directly by reinforcement learning without internal models, the model-based process generates in the brain an internal model of the environment (such as cognitive map; Tolman, 1948), grasps the relationship among states in the environment, and predicts rewards depending on these relationships (Glascher et al., 2010). To segregate the model-free and model-based processes, state transition tasks were used in several studies (Glascher et al., 2010; Daw et al., 2011; Doll et al., 2012; Lee et al., 2014; Deserno et al., 2015). For example, in a task with a structure that has a SR relationship as shown in Figure 1A, we may predict different responses depending on whether the model-free strategy (Figure 1B, left) or the model-based strategy (Figure 1B, right) is adopted. In the state transition task, state prediction error (SPE) can be calculated for each choice because transitions between choices are determined stochastically. Glascher et al. (2010) showed that reward prediction error (RPE) calculated from the model-free process is represented in the striatum and that SPE is represented in the LPFC. Because SPE cannot be calculated from the model-free process alone but requires the model-based process instead, it seems the state transition task is useful for separating the model-free and model-based processes in the striatum and LPFC. Daw et al. (2011) also showed that a RPE calculated from the model-free process was represented in the striatum with a state transition task in Figure 1. They additionally showed a RPE calculated from the model-based process which happened to be represented in the striatum rather than the LPFC. Based on these results, Daw suggested that this task can separate the model-free and model-based processes but cannot separate the striatal function and LPFC functions. This proposal has been further supported by several studies (Wunderlich et al., 2012; Lee et al., 2014; Walsh and Anderson, 2014; Deserno et al., 2015). Therefore, the hypothesis that the differences in the function of reward prediction in these two areas corresponds to the difference between reward prediction using model-free and model-based processes appears dubious.

Here, we attempt to dissociate the reward prediction functions of the striatum and the LPFC by focusing on two elements. The first is the existence/non-existence of information on transition among environmental states. This has long been discussed in relation to rule-based behavior and higher-order conditioning and many studies have shown the importance of the LPFC for such types of behavior (Sakagami and Niki, 1994; White and Wise, 1999; Hoshi et al., 2000; Wallis et al., 2001; Amemori and Sawaguchi, 2006; Han et al., 2009; Vallentin et al., 2012). The second element we consider is whether and how the LPFC and striatum use subjects’ experience to predict reward. This has a history of debate in relation to syllogism, transitive inference, categorical inference, and so forth (McGonigle and Chalmers, 1977; Blaisdell et al., 2006; Murphy et al., 2008). It has been argued that the model-free reinforcement learning process, which is believed to be performed in the nigro-striatal circuit, requires direct experience of obtaining reward for reward prediction (Schultz et al., 1997; Daw et al., 2005; Doya, 2008). On the contrary, it has been argued that the LPFC may integrate several pieces of fragmentary information to associate a stimulus with a future event without learning their interrelations directly (Miller, 2000; Duncan, 2001; Pan et al., 2008, 2014; Pan and Sakagami, 2012). However, the amount of research that has attempted to directly investigate these ideas in regards to striatum and LPFC function remains meager. Therefore, we shall discuss here whether experimental results and the difference in the functions of the prefrontal cortex and striatum are consistent with the two elements proposed above.

## The Sequential Paired-Association Task with an Asymmetric Reward Schedule

**FIGURE 2:**
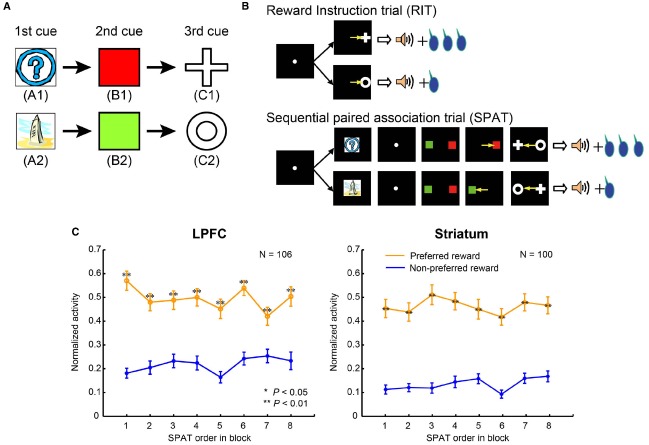
**The sequential paired-association task and neuronal activities in the LPFC and striatum. (A)** Two stimulus–stimulus associative sequences were learned by monkeys. These six stimuli were denoted as “old stimuli”; the monkeys extensively experienced them with different amounts of rewards. **(B)** An asymmetric reward schedule was used in each block in which RITs were presented first and then followed by SPATs. The stimulus-reward contingency was the same in RITs and SPATs within each block, but this was randomized between blocks. The yellow arrows indicate saccadic eye movements and were not actually shown in the experiment. **(C)** Population activity of reward neurons to the old stimuli (A1 and A2) when they were presented as the first cue in SPATs. The left column shows neuronal activity in the LPFC, and the right column shows the activity in the striatum. The x-axis in the figure indicates SPAT order in the block directly after RITs. The normalized activity of each neuron was sorted into the preferred reward condition (orange curves) and the non-preferred reward condition (blue curves), and this was averaged across the population of reward neurons. Statistical significance was determined by Mann–Whitney U test, **P < 0.01. Error bars indicate SEM.

To clarify the difference in the reward predictive functions in the LPFC and striatum, Pan et al. (2008) developed a reward inference task. In this task, the monkey subjects first learned two stimulus–stimulus association sequences (here denoted: A1→B1→C1 and A2→B2→C2, where A1, B1, C1, A2, B2, and C2 were six different visual stimuli; Figure 2A). These were learned in sequential paired-association trials (SPATs) with a symmetrical reward schedule (Pan et al., 2008). After having mastered the task, the monkeys were then taught an asymmetric reward schedule using reward instruction trials (RITs), in which one stimulus (C1 or C2) was paired with a large reward (0.4 ml of water) and the other stimulus (C2 or C1) with a small reward (0.2 ml of water). In behavioral and single-unit recording sessions, RITs were followed by SPATs within each block (Figure 2B). In the SPATs, the amount of reward received at the end of correct trials was consistent with that of the RITs from the same block: if C1 had been paired with the large reward, and C2 with the small reward in RITs, then in the subsequent SPATs the sequence A1→B1→C1 would lead to the larger reward, while the sequence A2→B2→C2 would lead to the smaller reward, and *vice versa*. Because in each block, subjects were taught in RITs whether C1 or C2 would be paired with the larger reward in the following SPATs, and because these associations changed randomly between blocks, experience from the previous block could not be effectively used to predict reward in the SPATs of the current block.

Pan et al. (2008) investigated whether monkeys would be able to transfer the reward information associated with C1 and C2 in the RITs to the first visual stimuli, A1 and A2, in the SPATs. Stimuli A1 and A2 were not directly paired with the different amounts of reward. However, if the monkeys could use both the SR relations (C with reward amount), and the stimulus–stimulus (A→B→C) associations, then after the RITs they should be able to predict reward amount at the time of the first stimulus presentation of A1 or A2 in a SPAT. On the contrary, if the monkeys just depended on the experience of SR relations from the previous block, their reward prediction should not necessarily be correct, particularly at the time of the first presentation of A1 or A2 in the first SPAT. Behaviorally, Pan et al. (2008) confirmed a significant decrease in performance in the first choice of the first SPAT (selection of B on the basis of A) when the trial used a sequence leading to smaller amount of reward (see Figure 1D in Pan et al., 2008). This shows that despite not yet receiving the reward itself, performance differed right from the first stimulus presentation depending on reward size (and therefore probably motivation). This indicates that monkeys were able to infer which reward condition they were currently experiencing right from the first stimulus presentation after reward instruction of C1 and C2.

Two different neuronal response patterns in the sequential paired-association task with an asymmetric reward schedule can be predicted based on the model-based and model-free learning processes. Using model-based learning processes (Daw et al., 2005), relevant brain areas should represent stimulus–stimulus associations acquired in the task in a tree-search manner, i.e., A1→B1→C1 and A2→B2→C2. Once C1 has been paired with the large reward in RITs and A1 is then presented in the SPAT as the first cue, the model-based system would search the tree-structure from A1 to B1, from B1 to C1 and from C1 to the large reward, and thereby predict that A1 would be associated with the large reward. In contrast, the model-free system does not store stimulus–stimulus associations; instead it saves a “cached” reward value associated with each stimulus (Daw et al., 2005). For example, when the brain experiences that in the current block the A1-sequence is paired with the large reward and the other sequence with the small reward, a larger value (e.g., 1) is assigned to the stimuli A1, B1, and C1, and a smaller value (e.g., 0) to the stimuli A2, B2, and C2. In the next block, the brain then learns that the stimulus C1 is paired with the small reward in RITs and the value of C1 is changed from 1 to 0, however the values of A1 and A2 remain yet unchanged. When A1 is then presented in SPATs, the model-free system would predict that it would lead to the large reward because A1 is still associated with the larger reward value. Overall, the model-based system is expected to predict reward information for the first stimulus (A1 or A2) on the basis of associations between the stimulus (C1 or C2) and reward acquired in RITs in the current block, while the model-free system is expected to predict reward information for the first stimulus on the basis of experience from the previous block. By considering the neuronal activity recorded from the LPFC and the striatum in the sequential paired-association task with the asymmetric reward schedule, we can verify whether the neurons in these areas use the model-based learning process or the model-free learning process.

## Ability to Use State Transition to Predict Reward

**FIGURE 3:**
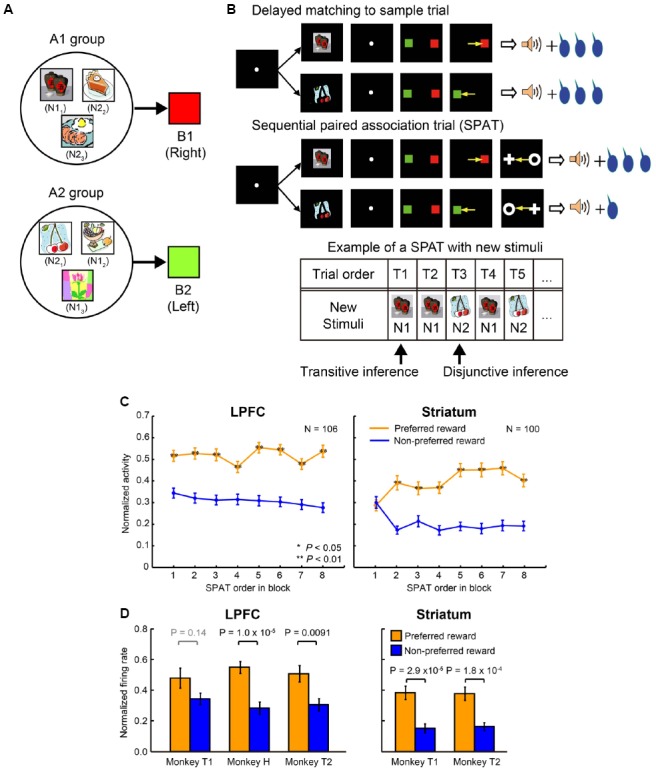
**Transitive and disjunctive reward inferences in the LPFC and striatum. (A)** Examples of new stimuli associated with the two color patches (B1 and B2). New stimuli that were associated with B1 were classified into the A1-group, and new stimuli that were associated with B2 were classified into the A2-group. **(B)** Reward inference in the sequential paired-association task with new stimuli. The monkeys learned new associations in a delayed matching-to-sample task in which the positions of B1 and B2 were fixed (e.g., B1 was always presented on the right side of the fixation spot, while B2 was always presented on the left side). The procedure of the asymmetric reward schedule is almost same as those shown in Figure 2B, except that a new stimulus were presented as the first cue instead of an old stimulus (A1 or A2) in SPATs. In the very first SPAT after reward instruction trials, the monkeys and neurons had no direct experience of new stimuli and asymmetric reward associations. The bottom panel shows an example new stimulus pair presented in a block. After RITs, one of the paired stimuli (N1) was presented as the first cue in the first (T1) and second (T2) SPATs. The other new stimulus of the pair (N2) was presented in the third SPAT (T3), and so on. If neurons could predict reward information for N1 in the very first SPAT (T1), one could say that these neurons have the ability of transitive reward inference (C1→reward and N1→B1→C1, so N1→reward). If neurons cannot predict reward information for N1 in T1 trials, but can infer reward information for N2 presented T2/T3 trials after directly experiencing the N1-reward contingency, one could say that these neurons have the ability of disjunctive reward inference. **(C)** Population activities of reward neurons to new stimuli as a function of SPAT order in the first block where new stimuli were presented for the first time. LPFC neurons discriminated the two reward conditions (preferred vs. non-preferred) from the very first SPAT (T1) after reward instruction trials (left column). Striatal neurons did not distinguish the preferred reward condition from the non-preferred reward condition in T1 trials. The orange curves represent the preferred reward condition, and the blue curves represent the non-preferred reward condition. Statistical significance was determined by Mann–Whitney U tests, *P < 0.05, **P < 0.01. Error bars indicate SEM. **(D)** Both the LPFC (left column) and striatal (right column) activities to the second new stimulus (N2) discriminated the reward conditions in T2/T3 trials after direct experience with the first new stimulus (N1) and with the reward contingency. The orange bars indicate the preferred reward condition and the blue bars indicate the non-preferred reward condition. Statistical significance was calculated by Wilcoxon rank sum test. Error bars indicate SEM.

Pan et al. (2008) recorded single-unit activity in the LPFC and striatum of monkeys performing this task (for recording sites, see Figures 3 and 4 in Pan et al., 2014). The majority of reward neurons, which were defined as showing differential averaged activity for stimuli indicating different amounts of reward, modulated their activity at the time of the first stimulus presentation in a SPAT (229/546 recorded neurons in the LPFC and 188/366 in the striatum). Results showed that LPFC neurons discriminated the large reward condition from the small reward condition right from the first SPATs (Figure 2C, left), indicating that LPFC neurons performed in a model-based manner. In addition to this, striatal neurons also distinguished the two reward conditions from the first SPATs in one block (Figure 2C, right), inconsistent with the predicted response pattern from model-free process. The findings that even striatal neurons could correctly predict rewards right from the first SPAT immediately after RITs indicate that striatal neurons also possess some information about state transition of stimuli in the SPAT task. Therefore, it is reasonable to suggest that the striatal neurons, in addition to the LPFC neurons, perform reward prediction in a model-based manner.

Model-based signals in the striatum have been found in both the state transition task and the sequential paired-association task (Daw et al., 2011; Lee et al., 2014; Pan et al., 2014), suggesting that the striatum may not simply use mode-free learning rules to predict reward. Neither of these two tasks could dissociate reward prediction functions in the LPFC and striatum. However, when we examined these two tasks carefully, we found subjects to have extensively experienced state transition, stimulus–stimulus and SR associations. Therefore, it is possible that striatal neurons simply utilize memorized relations (experiences) to predict reward. The behavior and neuronal activity patterns that can be predicted based on memorized experiences should be similar to results based on the model-based strategy. For example, the sequential paired-association task with an asymmetric reward schedule was repeatedly performed using six fixed stimuli: A1, B1, C1, A2, B2, and C2. There were four conditioned SR associations in the task, (1) C1→LR (large reward), A1→LR and A2→SR (small reward); (2) C1→SR, A1→SR and A2→LR; (3) C2→LR, A1→SR and A2→LR; (4) C2→SR, A1→LR and A2→SR. The monkeys extensively experienced each of these associations. If the monkeys and neurons memorized each of the conditioned SR associations, it would be easy for them to determine which stimulus (A1 or A2) would be paired with a large reward after reward instruction with C1 or C2. In that case, the responses of the LPFC and striatal neurons could be explained by the reward prediction based on memorized experiences. Some studies have shown that the activity of dopamine neurons could be modulated by memorized experiences of state transitions and SR associations to represent RPE and play a role in reward learning in the striatum (Nakahara et al., 2004; Bromberg-Martin et al., 2010; Enomoto et al., 2011). These results together suggest that in order to disassociate reward prediction functions in the LPFC and striatum, it is important to consider the effect of experience with reward-stimulus associations in the task. To investigate this issue, Pan et al. (2014) conducted a study where the monkeys were required to predict reward for a stimulus without any direct reward experience.

## Ability to Predict Reward Without Experience

Pan et al. (2014) conducted an experiment where session-unique stimuli were introduced into the above task and tested whether the monkey’s behavior and LPFC and striatal neurons could carry out similar reward prediction with newly introduced stimuli. The monkeys were trained to learn new stimulus associations in a delayed matching-to-sample task with a symmetric reward schedule (Figure 3A). The new stimuli were learned to be associated with one of the two color patches (B1 or B2). These newly learned stimuli are referred to as “new stimuli,” while the stimuli A1, B1, C1, A2, B2, and C2 are referred to as “old stimuli.” In total, the monkeys learned 924 new stimuli (462 new stimulus pairs) to be associated with the color patches (B1 or B2). For ease of explanation, the new stimulus that was randomly selected from each pair to be presented in the very first SPAT of the relevant block shall be referred to as N1, and the second new stimulus of each pair shall be referred to as N2; however it is important to note that there is not simply one N1 stimulus but 462 N1 stimuli, and the same for N2 stimuli (N1_1_–N1_462_ and N2_1_–N2_462_). The newly learned stimuli were classified into two groups according to the old stimuli that they were associated with. The new stimuli associated with B1 were classified into the A1-group and the new stimuli associated with B2 were classified into the A2-group (A1, B1, and C1 belonged to A1 group and A2, B2, and C2 to A2 group; Figure 3A). Up to this point, the monkeys had experienced no direct associations between new stimuli and C1 or C2, and also no information about the asymmetric reward schedule with respect to the new stimuli (Figure 3B, upper panel). After having fully acquired the new associations, the monkeys performed the reward instructed sequential paired-association task with the new stimuli (Figure 3B, middle panel). This was identical to the reward instructed SPATs with old stimuli except that in these SPATs a newly learned stimulus was presented as the first cue instead of an old stimulus (A1 or A2). Behaviorally, the monkeys showed significantly higher performance when first choosing from new stimuli in the large than in the small reward trials (see Figure 2 in Pan et al., 2014). This indicates that the monkeys correctly predicted the reward information for the first new stimulus (N1 or N2) that was presented in SPATs based on the reward information associated with C1 or C2 in RITs, without the requirement of direct associations between the reward information and the new stimuli.

Reward-related neurons from the LPFC and striatum were recorded while monkeys performed the reward instructed SPAT task with new stimuli. Almost all the reward-related neurons in the LPFC and striatum, which showed reward differential activity for A1 and A2 in SPATs with old stimuli, showed similar reward differential activity with new stimuli (at least on average). When Pan et al. (2008) concentrated on the single-unit activity to the first new stimulus (N1; independent of the stimulus group it belonged to, Figure 3B, bottom panel) in the very first SPAT just after the RITs, reward-related neurons in the striatum did not show differential activity regardless of whether N1 predicated large or small reward (Figure 3C, right), however reward-related neurons in the LPFC did (Figure 3C, left). The striatal reward-related neurons did discriminate the two reward conditions from the second presentation of N1. These results seem to show that the striatum needs direct experience of SR associations to predict reward. However, when Pan et al. (2008) looked at neuronal activity to the second new stimulus (N2) when it was first presented in the SPAT (after one or two presentations of N1), even the striatal neurons could show reward differential activity (Figure 3D). This result indicates that in a new stimulus pair (N1 and N2) striatal neurons are able to infer the reward amount of N2 (after receiving reward information about N1) without direct experience of which reward amount would follow N2. This type of function is called a disjunctive inference (Johnson-Laird et al., 1992).

Lateral prefrontal cortex neurons could predict the reward amount of a new stimulus from the very first SPAT (just after the RITs). This result cannot be explained by the reward prediction based on memorized experiences because at this point the monkeys had no past experience of the appropriate SR assignment. Therefore, this result indicates that neurons in the LPFC had the ability to combine the results of two associations to predict future outcomes. This ability is called a transitive inference. In this experiment LPFC neurons combined the association of the new stimuli with C (through B), with the association of C with reward size, to predict the reward amount. Striatal neurons were unable to combine these stimulus–stimulus and SR associations to predict reward. Instead striatal neurons could predict the reward amount (e.g., small) of the second new stimulus (N2) from a pair after directly experiencing the alternative stimulus (N1) with the alternative amount of reward (e.g., large). This result indicates that the striatal neurons can perform disjunctive inferences. Congruent with this finding, Bromberg-Martin et al. (2010) reported that dopamine neurons, which have a close relationship with reward prediction in the striatum, are able to use disjunctive inferences to generate RPE signals. Furthermore, disjunctive inference is similar to a key function used for establishing the model-based process in the state transition task performed by Daw et al. (2011). The evidence that the nigro-striatal network is involved in reward prediction via disjunctive inference indicates that the model-based vs. model-free process distinction is not simply equivalent to dissociation in LPFC and striatal functions.

## Reward Inference by Abstract Neural Code

We further investigated why it may be that LPFC neurons can perform transitive reward inference while striatal neurons cannot. Pan et al. (2008) found a subgroup of reward neurons (SR type) in the LPFC that showed differential reward activity for only one of the first stimuli (A1 or A2). Originally, Pan et al. (2008) used an ABC sequence (A1→B1→C1 and A2→B2→C2) for recording and looked at the activity pattern to stimulus A (A1 or A2). However, by investigating only this sequence, they could not tell whether SR type neurons reflect categorical information or whether they simply reflect visual properties of the first cues. To address this, Pan et al recorded activity of SR neurons in the LPFC with another two sequential associations: the BCA sequence (B1→C1→A1 and B2→C2→A2) and the CAB sequence (C1→A1→B1 and C2→A2→B2). The majority of SR neurons showed reward-differential activity only for a group of relevant visual stimuli (e.g., A1, B1 and C1, or A2, B2 and C2; Figure 4A). This tendency was confirmed by the population activity of SR neurons (Figure 4B). Therefore, these neurons (hereby referred to as “category-reward” neurons) likely coded both the category information of visual stimuli (either A1 or A2 group), and reward information (either large or small), simultaneously.

**FIGURE 4:**
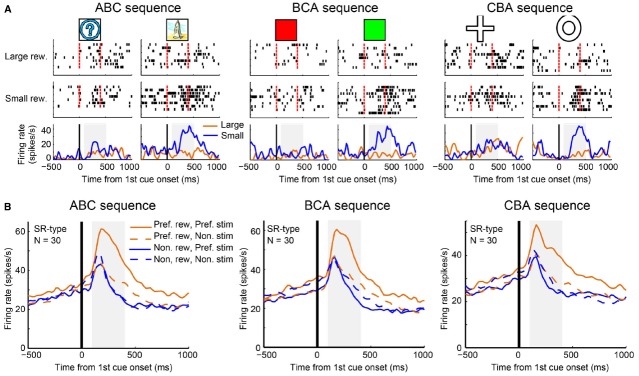
**Example activity of a stimulus-reward neuron and population activities. (A)** Single-unit activity of a stimulus-reward neuron for three sequences: ABC (A1 vs. A2), BCA (B1 vs. B2), and CAB (C1 vs. C2) sequences. In each sequence, all trials were sorted by four conditions—the first cue stimulus (i.e., A1 vs. A2) and the two reward conditions (larger reward vs. small reward)—and aligned with the first cue onset. In the raster-grams, red ticks mark the onset and offset of the first cue. In the histograms, the orange curves represent data from large-reward trials and the blue curves represent data from small-reward trials. **(B)** The population histograms of stimulus-reward neurons for three sequences. Trials of each stimulus-reward type cell were sorted by four conditions: preferred reward condition and preferred stimulus (solid orange curves), preferred reward condition and non-preferred stimulus (dashed orange curves), non-preferred reward condition and preferred stimulus (solid blue curves), and non-preferred reward condition and non-preferred stimulus (dashed blue curves).

In related literature, many studies have reported that neurons in the LPFC code categorical information (Freedman et al., 2001; Shima et al., 2007; Meyers et al., 2008; Cromer et al., 2010; Seger and Miller, 2010). Sakagami and Tsutsui (1999) trained monkeys to make a go response to, for example green and purple colors, and a no-go response to, for example red and yellow colors (Sakagami and Tsutsui, 1999). Many neurons in the ventrolateral PFC showed go/no-go differential activity based on color (color go/no-go neurons). A majority of them also showed color grouping. For example if a neuron showed differentially increased activity to green, then the same neuron tended to show the same activity pattern to purple, while if a neuron increased activity to red, then the same neuron tended to show the same activity pattern to yellow. Activity of these neurons did not simply reflect go/no-go discrimination because the same neurons did not show differential activity when the monkeys performed the same go/no-go task with motion cues. As the LPFC is located between sensory output areas and motor execution areas, the task of the LPFC is likely related to the conversion of sensory information to motor information (Sakagami and Pan, 2007). If the area has several hierarchical stages for this process, it is natural that some neurons in the LPFC should code both sensory and motor information in a manner similar to the “abstract” coding seen in both reward-category neurons and in the color go/no-go neurons. In support of this idea, Shima et al. (2007) found “motor-category” neurons in the LPFC.

It is an interesting question to ask whether or not striatal neurons encode category information. Evidence against this was found in a recent study (Antzoulatos and Miller, 2011). In this study, Antzoulatos and Miller (2011, 2014) compared neuronal activity patterns for the LPFC and striatum during a dot-based shape category learning task in which the monkeys learned to associate stimuli from one category with a right saccade and stimuli from the other category with a left saccade. In each recorded session, two novel dot-pattern prototypes (two categories) were introduced. In the first block, a single stimulus per category was presented and the monkeys learned the relevant stimulus-response associations. In following blocks, more and more new stimuli were added. The monkeys were unable to learn each stimulus-response association; instead, they learned category-response associations. It was found that striatal neurons represented stimulus-response association in the early learning stage. In the late learning or category performing stage, LPFC neurons encoded category-response associations but striatal neurons did not represent such category information. These results suggest that striatal neurons do not classify new category members into a group or represent their category information.

In the sequential paired-association task of Pan et al. (2008) the monkeys might have, through extensive training, grouped stimuli requiring the same response together as a functional category according to intended behavior. Some LPFC neurons appeared to represent category information of those associated stimuli. It is known that LPFC neurons also receive reward information from the OFC and subcortical areas, such as the striatum, amygdala, and dopaminergic neurons in the midbrain (Schultz, 2000; Wallis and Miller, 2003; Sakagami and Watanabe, 2007). Therefore, some LPFC neurons involved in categorization might also receive reward information and thereby function as category-reward neurons. In the sequential paired-association task with new stimuli, the monkeys were unable to rely on rote memory to predict the amount of reward for the new stimuli because they had not yet been directly taught associations between the new stimuli and reward. Effectively, the monkeys had to integrate independently acquired associations to infer the reward value of new stimuli. The category-reward neurons may be what the brain uses to fulfill this integration function. Each member from the preferred category was found to evoke similar response patterns in category-reward neurons; this processing may be the way in which relations between reward and each member in the same category were established. If a newly introduced stimulus is a functional member of a given category, then the category information of the new stimulus and the reward information acquired in RITs could together activate the category-reward neurons. This activity of the category-reward neurons may allow reward neurons in the LPFC to infer the reward information of the new stimuli. The striatal neurons were unable to predict reward for the first new stimuli presented in the first SPATs. As shown above, the striatal neurons were not able to use this categorical code approach. Instead they likely used memorized experiences to know which reward N2 must be associated with after directly experiencing the alternative reward in association with N1. Our suggestion that the LPFC but not the striatum may be capable of categorical coding is reinforced by the finding that LPFC neurons showed categorical related activity whereas striatal neurons showed response related activity in a category learning task (Antzoulatos and Miller, 2011, 2014). Overall, reward prediction neurons in the LPFC, but not those in the striatum, were able to predict the reward amount of newly introduced stimuli at their first presentation in SPATs possibly because the LPFC is capable of categorical coding whereas the striatum is not (Figure 5).

**FIGURE 5:**
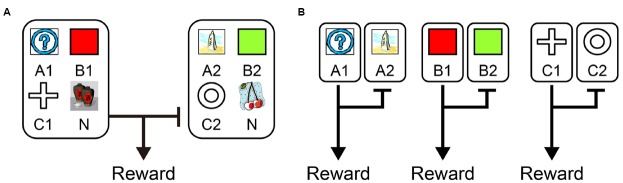
**Schematic drawing of the function of LPFC and striatal neurons. (A)** LPFC neurons can use knowledge of categorical relationships to predict reward. **(B)** Striatal neurons can code the individual associations between stimuli and reward.

## Discussion and Conclusion

Here, we suggest that LPFC neurons can perform the categorization process. The categorization process is regarded as the process utilized to determine which things belong together. In a related study three types of categories were proposed: the “perceptual category,” the “relational category,” and the “association category” (Zentall et al., 2002). The categorization process we discussed here is likely to have been of the associative type, in which shared functions of the stimuli are important instead of the physical properties of them. The LPFC encodes associative categorization (Pan et al., 2008), and may utilize it as a model to simulate or predict reward information for both well learned old stimuli and newly introduced stimuli. This process does not require the animals and neurons to directly experience associations between stimuli and reward. The striatum did not represent the category information as a model; instead, it might have stored paired stimuli-reward information to predict reward after directly experiencing the association between one stimulus of a pair and its associated reward. The functional difference between the LPFC and striatum might not simply rely on model-based vs. model-free learning rules. It might instead rely on whether and how the two areas integrate experiences with current task state information and use different strategies to predict reward.

The probabilistic state transition task performed by Daw et al. (2011) and the sequential paired-association task with old stimuli by Pan et al. (2008) both consist of several sequences of stimuli or actions. In both tasks, to obtain reward at the end of trial, it is necessary to assign the potential reward to early stimuli or actions properly. This credit assignment program has been discussed within the framework of explicit/implicit learning processes (Fu and Anderson, 2008). Fu and Anderson (2008) performed a state transition task similar to the task performed by Daw et al. (2011) and showed that the subjects displayed faster state learning when explicit memory was dominantly relied upon and that implicit reinforcement learning required state information. Therefore, these explicit/implicit learning processes are presumably related to the model-based/model-free learning processes. On the other hand, in Pan et al.’s (2008) sequential paired-association task with new stimuli, the monkeys’ behaviors and LPFC activity showed reward related activities without the direct experience of obtaining reward after presentations of these new stimuli. This result indicates that reward value can be assigned to the stimuli without experience. It is possible to explain this by suggesting that reward value is assigned to abstract information, such as the functional category, rather than to representation of the individual stimulus. Therefore, experience of reward with any member of a category causes reward to be assigned to this whole category, thereby making it possible for one to be able predict reward for another member of the same category without direct experience.

Where could the model-free learning process be performed in the brain? We believe that the model-free reinforcement learning process is the basis of the reward related learning. Furthermore, a lot of studies support the idea of the existence of a circuit for reinforcement learning in the nigro-striatal circuit (Schultz et al., 1997; Daw et al., 2005; Doya, 2008). Therefore, the model-free learning process is likely performed in parts of the striatum. However, even if the nigro-striatal circuit simply performs the model-free reinforce learning process, when it receives signals calculated in the prefrontal cortex with the model-based process, its activity then appears to perform the model-based learning process. In other words, the learning process in the striatum depends on the signal sent to the striatum. Recently, it was proposed that the LPFC works as an arbitrator of model-based and model-free strategies (Lee et al., 2014). Therefore, it is possible that the LPFC controls the signal sent to the striatum and allocates the degree of control over behavior determined by model-based and model-free systems. Nonetheless, the degree to which we can understand the learning process performed in the striatum based simply on subjects’ behaviors and neural activities may be limited. To precisely understand the learning process performed in the striatum, it would be necessary to reveal the signal processing mechanism in the striatum using methods which can examine the circuit mechanism directly, i.e., optogenetics and the DREADD (Designer Receptors Exclusively Activated by Designer Drugs) system.

There remains no doubt about the existence of the model-based learning process in the LPFC. Here, we extend this idea by specifically proposing the existence of abstraction or categorization in the LPFC. While this can explain the SR associations found in the SPAT task, more precise neurophysiological research is required to explain the model-based learning process in the state transition task. Furthermore, it is suggested that the LPFC also contributes to other complex cognitive processes which are involved in the model-based system (Yoshida et al., 2010; Donoso et al., 2014). Further study is necessary to extend understanding of the specific function of the LPFC in model-based learning processes.

In conclusion, to clarify the functional differences in reward prediction between the LPFC and striatum in the monkey brain, we compared activity patterns of neurons in these two areas mainly from studies using a sequential paired association task with an asymmetric reward schedule. To predict reward, both LPFC and striatal neurons were able to use knowledge about state transitions. LPFC neurons could predict reward via transitive inference and striatal neurons could predict reward via disjunctive inference even in previously unexperienced situations. These results suggest the existence of a model-based system in both the LPFC and the striatum. These results also indicate that the model-based vs. model-free hypothesis is not sufficient to explain the functional difference between the LPFC and striatum. Instead, the difference seems to be that the LPFC neurons can utilize abstract code (in this case stimulus categorization; Figure 5A) to associate a stimulus with a reward, whereas while the striatal neurons can code the individual associations between a stimulus and a reward (or sequence of stimuli-reward, Figure 5B), they cannot use abstract code.

### Conflict of Interest Statement

The authors declare that the research was conducted in the absence of any commercial or financial relationships that could be construed as a potential conflict of interest.
